# Supplementary material for: Sleep Disorders in Pediatric Patients Affected by Neurofibromatosis Type 1: Reports of a Questionnaire and an Apple Watch Sleep Assessment
Source: Biomedicines. 2025 Apr 8;13(4):907. doi: 10.3390/biomedicines13040907 (PMC12024556; doi:10.3390/biomedicines13040907)
Supplement: Supplementary file 1 [file biomedicines-13-00907-s001.zip › biomedicines-3466809-supplementary.pdf]

EXPERIMENTAL THESIS ON PAEDIATRIC SLEEP DISORDERS

GRADUATED STUDENT: ALESSIA MIGLIORE

QUESTIONNAIRE

Anonymous survey in order to obtain clinical data for the elaboration of an experimental thesis. It will take a maximum of 5 minutes. Thank you very much for your cooperation!

Please indicate the gender of your child:

☐ Male

☐ Female

Please indicate the age of your child:

....

Please state your child's weight:

....

Please state your child's height:

....

Does your son/daughter suffer from any of the diseases listed below?

☐ Respiratory diseases (asthmatic bronchitis, asthma, allergic rhinitis or other)

☐ Digestive system diseases (gastroesophageal reflux, food intolerances/allergies, chronic inflammatory bowel disease, celiac disease or other)

☐ Neurological diseases (epilepsy, neuro-skin diseases, headache or other)

☐ Metabolic diseases (diabetes, hypertriglyceridemia, phenylketonuria, mucopolysaccharidosis or other)

☐ Haematological diseases (anaemia, thrombocytopenia or other)

☐ Neuropsychiatric illnesses (ADHD, autism or other)

☐ Not affected by any pathology

☐ Other

Please specify the pathology/ies below:

....

Does your child take medication for this/these pathology/ies?

☐ Yes

☐ No

If yes, which ones?

....

Does your son/daughter have certain habits before going to bed to promote the onset of sleep? (Dependence on certain actions, circumstances, or objects to initiate sleep).

☐ Uses electronic devices (watches TV, plays video games, uses tablets or mobile phones)

☐ Is rocked

☐ Is breastfed

☐ Spends time in bed with parents

☐ None of the above

Does your child engage in behaviour with the aim of staying awake longer and not going to bed?

☐ Yes, continues to play or prolong the activity he/she was doing, cries or throws tantrums

☐ No

How long does it take your child to fall asleep once in bed?

☐ 0-15 minutes

☐ 15-30 minutes

☐ More than 30 minutes

Does your son/daughter experience any of the following complaints during falling asleep and/or waking up?

☐ Temporary inability to move or speak, lasting a few seconds or minutes (sleep paralysis)

☐ Hallucinations

☐ Starts sleeping well after midnight with difficulty waking up (delayed sleep syndrome)

☐ Early night awakenings without the possibility of falling asleep again (04:00-06:00)

☐ None of the above

If yes, how often do the episodes occur?

☐ Rarely (1-2 times per month)

☐ Frequently (once a week)

☐ Very frequently (more than 3 times per week)

Does your son/daughter experience any of the following complaints during sleep?

☐ Confusional awakening

☐ Frequent nocturnal awakenings

☐ Recurring nightmares

☐ Sleepwalking

☐ Experiences a feeling of "pain", "tingling" in the legs accompanied by the need to move and kick (restless leg syndrome) or periodically moves limbs

☐ Cramps in the legs and feet during sleep that cause them to wake up

☐ Bruxism (involuntary teeth grinding)

☐ Night sweats (hyperhidrosis)

☐ None of the above

If yes, how often do the episodes occur?
☐ Rarely (1-2 times per month)
☐ Frequently (once a week)
☐ Very frequently (more than 3 times a week)

Does your son/daughter have any of the following respiratory sleep disorders?
☐ Difficulty breathing
☐ Heavy breathing
☐ Snoring
☐ Sleep apneas
☐ Sudden awakenings with choking sensation associated with profuse sweating
☐ None of the above

If yes, how often do the episodes occur?
☐ Rarely (1-2 times per month)
☐ Frequently (once a week)
☐ Very frequently (more than 3 times a week)

Do the complaints occur consistently over time?
☐ Yes, regularly
☐ No, alternating with periods of remission of the disorder

Following the presentation of the sleep disorder, do you notice the following conditions occurring during the course of the day?
☐ Daytime sleepiness
☐ Headache
☐ Impaired cognitive performance
☐ None of the above

When your son/daughter does not sleep well, do you notice any behavioural changes in him/her?
☐ Decreased school performance (difficulty maintaining concentration)
☐ Episodes of falling asleep in class
☐ Irritability and/or aggressiveness
☐ Mood swings
☐ Is more anxious than normal
Tends to be sad
☐ None of the above

When your son/daughter does not sleep, how do you (mother) feel?
☐ I am irritable more easily

☐ I concentrate less at home and at work

☐ I have anxiety/panic attacks

☐ I feel sadder than usual

☐ None of the above (I can cope)

When your son/daughter does not sleep, how do you (father) feel?

☐ I am irritable more easily

☐ I concentrate less at home and at work

☐ I have anxiety/panic attacks

☐ I feel sadder than usual

☐ None of the above (I can cope)

Does your child sleep during the afternoon?

☐ Yes, 3 or more times a week

☐ Yes, less than 3 times a week

☐ No/rarely

If he/she sleeps during the afternoon, how long is the nap?

☐ 10-30 minutes

☐ 30-60 minutes (half an hour to an hour)

☐ 60-120 minutes (1-2 hours)

☐ More than 120 minutes (2 hours or more)

Does your son/daughter consume drinks containing caffeine during the afternoon/evening?

☐ Yes (Coca Cola or other)

☐ No

To treat sleep disorders, does your son/daughter take melatonin?

☐ Yes, and it works well

☐ Yes, but it does not seem to work

☐ No

Does your son/daughter take medication to treat sleep disorders?

☐ Yes

☐ No

If yes, which ones?

....
